# Imposing Compulsory Rugby Union on Schoolchildren: An Analysis of English State-Funded Secondary Schools

**DOI:** 10.3389/fspor.2022.784103

**Published:** 2022-07-07

**Authors:** Adam John White, John Batten, Nathan E. Howarth, Rory Magrath, Joe Piggin, Pete Millward, Keith D. Parry, Melanie Lang, Rachael Bullingham, Alan J. Pearce, Luis Morales, Gary Turner, Connor Tyler Humphries, Jack Hardwicke, Eric Anderson, Graham Kirkwood, Allyson Pollock

**Affiliations:** ^1^Department of Sport, Health Sciences and Social Work, Oxford Brookes University, Oxford, United Kingdom; ^2^Warwick Medical School, University of Warwick, Coventry, United Kingdom; ^3^Concussion Legacy Foundation UK, Cheltenham, United Kingdom; ^4^Sport and Event Management, Bournemouth University, Poole, United Kingdom; ^5^School of Sport, Health and Community, University of Winchester, Winchester, United Kingdom; ^6^Faculty of Sport, Health and Social Sciences, Southampton Solent University, Southampton, United Kingdom; ^7^School of Sport, Exercise and Heath Sciences, Loughborough University, Loughborough, United Kingdom; ^8^School of Humanities and Social Sciences, Liverpool John Moores University, Liverpool, United Kingdom; ^9^Department of History, Geography and Social Sciences, Edge Hill University, Ormskirk, United Kingdom; ^10^School of Sport and Exercise, Human Services & Sport, University of Gloucestershire, Cheltenham, United Kingdom; ^11^School of Allied Heath, La Trobe University, Melbourne, VIC, Australia; ^12^Centre for Physical Activity and Life Sciences, University of Northampton, Northampton, United Kingdom; ^13^Population Health Sciences Institute, Newcastle University, Newcastle upon Tyne, United Kingdom

**Keywords:** injury, risk, safeguarding, physical education (PE), curriculum, risk acceptability, acceptance, school sport

## Abstract

**Objective:**

To establish the extent to which Rugby Union was a compulsory physical education activity in state-funded secondary schools in England and to understand the views of Subject Leaders for Physical Education with respect to injury risk.

**Method:**

A cross-sectional research study using data obtained under the Freedom of Information Act (2000) from 288 state-funded secondary schools.

**Results:**

Rugby Union was delivered in 81% (*n* = 234 of 288) of state-funded secondary school physical education curricula, including 83% (*n* = 229 of 275) of state-funded secondary school boys' and 54% (*n* = 151 of 282) of girls' physical education curricular. Rugby Union was compulsory in 91% (*n* = 208 of 229) of state-funded secondary schools that delivered it as part of the boys' physical education curriculum and 54% (*n* = 82 of 151) of state-funded secondary schools that delivered contact Rugby Union as part of the girls' physical education curriculum. Subject Leaders for Physical Education also perceived Rugby Union to have the highest risk of harm of the activities they delivered in their school physical education curriculum.

**Conclusion:**

Notwithstanding discussions of appropriate measures (i.e., mandatory concussion training, Rugby Union specific qualifications and CPD) to reduce injury risk, it is recommended that Rugby Union should not be a compulsory activity given that it has a perceived high risk of injury and is an unnecessary risk for children in physical education.

## Introduction

Community amateur adult Rugby Union is claimed to have a “high risk” of injury compared to other sports (Roberts et al., 2013). While there is limited epidemiological data on the school and youth context (including physical education and after-school clubs / matches) and community youth settings, where data is available, it shows a comparable risk of concussion between the adult community game and the schoolboy game (Viviers et al., 2018).^1^

Youth Rugby Union research from the community, academy and independent school settings (Haseler et al., 2010; Palmer-Green et al., 2013) has found most time-loss injuries to occur in the tackle. In line with this research, a recent epidemiological study of 1st XV school teams found 37% of 825 children suffered a time-loss injury over the course of a season (Archbold et al., 2017). Almost half (49%) of those injured required 28 or more days away from play, with around two-thirds (63%) of injuries occurring in the tackle phase. 19% of injuries (*n* = 81) were concussions (Archbold et al., 2017).

Other research (Kirkwood et al., 2019) has found that the three main sports (in order of frequency) that resulted in sport-related hospital admissions for males under the age of 19 years were football (soccer), Rugby Union, and Rugby League. Likewise, Abernethy and MacAuley (2003) found contact rugby, including school games, physical education classes and competitive school matches, to be responsible for 44% of school-sport injuries that required Accident and Emergency department attendance.

While acknowledging the different contexts, the findings of the above research and other literature (Freitag et al., 2015; Kirkwood et al., 2015) - which identify a high(er) risk of injury from contact rugby compared to other sports frequently played in school physical education (Association for Physical Education, 2016) - are in line with the call by academics and physicians for the UK Government to remove the tackle from rugby in school physical education (Pollock et al., 2017). Since there is no legal requirement for tackling in school physical education, coupled with no evidenced benefits of tackling for the physical and psycho-social health of participants (Pollock, 2014; Pollock et al., 2017), the heightened risk of injury associated with tackling is argued to be unnecessary for children in the school physical education context (White et al., 2018). Non-contact codes of rugby, such as touch or tag, might be played instead.

Some academics disagree (e.g., Tucker et al., 2016; Quarrie et al., 2017). Fuller (2007) has stated that ‘a high level of risk does not make a risk unacceptable *per se*; people [adults] will accept risks that are taken on a voluntary basis that are up to 1,000 times higher than risks taken on a non-voluntary basis' and on that basis voluntary participation with informed consent is essential (Fuller, 2007; White and Robinson, 2018). Unfortunately, however, injury data across sports on school children, particularly in the compulsory physical education context are not routinely collected, analyzed and/or disseminated by any authority.

In the UK Independent School context i.e., those that are fee-charging and not state-funded, Nyiri (2015) found that 77% of schools made Rugby Union compulsory. Yet, no such data exists in state-funded secondary school settings, which are often less well-resourced in comparison. In addition, Subject Leaders for Physical Education have not been consulted about injury risk – despite being the people at the forefront of delivery. Therefore, the purpose of this study was to (a) establish the extent to which Rugby Union was made a compulsory activity in state-funded secondary schools, and (b) understand which activities were perceived by Subject Leaders for Physical Education as having the highest risk of injury.

## Methods

This cross-sectional research study obtained data from state-funded secondary schools under the Freedom of Information Act 2000 (FOIA) between 9^th^ January 2017 and the 21^st^ July 2017. These schools – including academies and free schools – are publicly funded institutions and, as such, are legally required by the FOIA to respond to requests for data, if in possession of the information requested, within 20 working days.

Specifically, schools were required to provide information on: (a) physical education and school sport activities offered both inside and outside of the curriculum; and (b) compulsory physical education and school sport activities. Here, all assessable activities that are defined by the Department of Education were presented to schools, including modified versions of sports, such as touch and tag rugby. Additionally, Subject Leaders could voluntarily respond to a question about which of the activities delivered was perceived to have the highest risk of harm.

Within England, there were 3,408 state-funded secondary schools that educated pupils aged between 11 and 16 years in the 2016–2017 academic year.^2^ Of the 48 geographical counties of England, all of the secondary schools from 11 (Cheshire, Cornwall, Dorset, Essex, Gloucestershire, Hertfordshire, Merseyside, Northamptonshire, Northumberland, Staffordshire, Suffolk) were pooled, resulting in a total of 788 eligible schools. These counties were randomly selected following a geographical split of metropolitan and rural areas from across the country. Schools were included if they were state-funded and taught Key-Stage 3 (age 11 to 13 years) and 4 (age 14 to 16 years).

In England, compulsory National Curriculum subjects at Key Stage 3 include: English, maths, science, history, geography, modern foreign languages, design and technology, art and design, music, physical education, citizenship, and computing. Similarly, at Key Stage 4 - where most pupils work toward national qualifications (e.g., GCSEs) - compulsory National Curriculum subjects are “core” (English, maths, science) and “foundation” (computing, physical education, citizenship) subjects. Thus, physical education is compulsory under the National Curriculum at all key stages; with the National Curriculum programmes of study outlining what should be taught at each key stage.

All of the schools were randomized using https://www.random.org/lists/ and the first 400 schools became the contacted sample. In determining the sample, we had to balance the competing demands of a representative sample with the resourcing challenges and time available to the research team. As such, we aimed to obtain a sample of ~10% of the national school population (factoring in some non-responses).

Initially, schools were sent a voluntary request for information by email, which included the purposes of the study. They were then emailed 20 working days later with a more formal FOIA request, as appropriate. After 20 more working days, further letters and follow-up emails were sent to schools, where necessary. Subject Leaders retained the option to (not) respond to questions about perceived injury risk throughout this process.

The majority of responses were submitted *via* a pre-populated online survey using onlinesurveys.ac.uk. This online survey captured information on school demographics (e.g., number of pupils, school type, Ofsted Rating, Free School Meals provision, number of teachers) and the physical education curriculum, including a list of all possible activities available to male and female students at different key stages. Any responses that were submitted by post were entered online upon receipt (*n* = 16). The amount of curriculum time (exposure) devoted to each activity was not collected and is a limitation of this study.

As part of data collection, Subject Leaders for Physical Education were also asked to identify which of the activities delivered in school had the highest perceived risk of injury. Subject Leaders for Physical Education are responsible for the localized development and implementation of the physical education curriculum in schools. Thus, in the absence of injury monitoring and epidemiological data, Subject Leaders are currently best placed to offer a reasoned view on the perception of injury risk in school physical education.

Ethical approval was obtained from the University of Winchester prior to data collection. Although not a requirement under the FOIA, school names were redacted to maintain anonymity. Of the 400 schools contacted, there were 296 responses (72% response rate), of which 8 data entries were duplicates. Duplicates were identified by school name and IP address. All duplicates were excluded from the study, leaving a total sample size of 288 schools or 8% of state-funded secondary schools in England which, based upon pupil numbers (*n* = 293,414), includes 9% of state-funded secondary school pupils nationally.

While data for school type is unavailable nationally, this sample was dominated by Academy Converters (*n* = 160), followed by Academy Sponsor-Led (*n* = 49), Community (*n* = 32), Foundation (*n* = 15), Voluntary Aided (*n* = 14), and other school types (*n* = 13). While all schools were state-funded, they had varying levels of flexibility with regard to the physical education curriculum; e.g., Academy Convertor schools can opt to ignore the National Curriculum for Physical Education, as long as they provide a broad and balanced curriculum that promotes the physical development of pupils.

The mean average school size, based upon the number of pupils at the school, was 1,018 pupils (SD = 461), while the national mean average school size was 946 pupils, showing this sample to include slightly larger schools than average. Based on the schools' latest Ofsted rating, 55 schools were classified as “outstanding” (19%), 168 schools were classified as “good” (58%), 31 schools were classified as “requires improvement” (11%), 8 schools were classified as “inadequate” (3%), with data unavailable for 26 schools (9%).

Most of the schools in this sample were co-educational (*n* = 269), with boys and girls taught together - except in physical education, whereby boys and girls are often taught separately. Indeed, while guidance about gender separation in mixed (co-educational) schools' states that schools should not generally separate pupils by sex, section 195 of the Equality Act 2010 contains an exception that permits single-sex sport participation. Yet, where separation by sex does exist it remains unlawful for a school to treat one group less favorably; e.g., by providing better resources for boys than girls. The sample in the present study also included some all-boy (*n* = 6) and all-girl (*n* = 13) schools. Overall, the sample included 275 schools that educated boys and 282 schools that educated girls.

## Results

### School Physical Education

The 10 most offered activities within state-funded secondary school physical education in the present sample were: athletics (*n* = 281, 97.57%), soccer (*n* = 279, 96.88%), fitness (*n* = 277, 96.18%), rounders (*n* = 272, 94.44%), netball (*n* = 266, 92.36%), basketball (*n* = 265, 92.01%), cricket (*n* = 262, 90.97%), badminton (*n* = 260, 90.28%), dance (*n* = 243, 84.38%) and Rugby Union (*n* = 234, 81.25%). The most offered activities for boys were: soccer (*n* = 267, 97%), athletics (*n* = 266, 96.7%), cricket (*n* = 261, 94.9%), fitness (*n* = 261, 94.9%), basketball (*n* = 254, 92.4%), badminton (*n* = 245, 89.1%), Rugby Union (*n* = 229, 83.3%), table tennis (*n* = 203, 73.8%), tennis (*n* = 199, 72.4%) and gymnastics (*n* = 193, 70.2%). The top 10 most offered activities for girls within physical education were: athletics (*n* = 270, 95.7%), fitness (*n* = 265, 94%), rounders (*n* = 263, 93.3%), netball (*n* = 262, 92.9%), soccer (*n* = 251, 89%), badminton (*n* = 246, 87.2%), dance (*n* = 237, 84%), basketball (*n* = 224, 79.4%), gymnastics (*n* = 216, 76.6%) and tennis (*n* = 209, 74.1%).

### Compulsory Activities

Data for those activities that were made compulsory as part of the physical education curriculum are presented in Table 1. For boys, activities included soccer (*n* = 227), athletics (*n* = 212), Rugby Union (*n* = 208), cricket (*n* = 196), fitness (*n* = 191), basketball (*n* = 174), badminton (*n* = 155), gymnastics (*n* = 140), tennis (*n* = 118), and rounders (*n* = 107). For girls, these activities included netball (*n* = 227), athletics (*n* = 207), rounders (*n* = 193), fitness (*n* = 190), dance (*n* = 159), soccer (*n* = 159), gymnastics (*n* = 158), badminton (*n* = 143), basketball (*n* = 133), and field hockey (*n* = 129).

**Table 1:** The number of schools that make activities compulsory by gender.

**Activity**	**Number schools that deliver (of 288 schools)**	**Males**			**Females**		
		**Number schools that deliver for males**	**Compulsory**		**Number schools that deliver for females**	**Compulsory**	
			***N***	**%**		***N***	**%**
Athletics	281	266	212	79.7	270	207	76.67
Badminton	260	245	155	63.27	246	143	58.13
Baseball	43	43	13	30.23	24	7	29.17
Basketball	265	254	174	68.5	224	133	59.38
Boxing	18	13	1	7.69	11	1	9.09
Canoeing	18	7	2	28.57	6	2	33.33
Cricket	262	261	196	75.1	205	114	55.61
Cycling	41	22	9	40.91	25	9	36
Dance	243	162	101	62.35	237	159	67.09
Dodgeball	190	177	72	40.68	177	67	37.85
Equestrian	28	2	1	50	2	1	50
Fitness	277	261	191	73.18	265	190	71.7
Gaelic Football	19	16	4	25	6	2	33.33
Gymnastics	227	193	140	72.54	216	158	73.15
Handball	183	170	87	51.18	158	70	44.3
Hockey	208	172	95	55.23	198	129	65.15
Judo	2	1	0	0	2	2	100
Netball	266	127	54	42.52	262	227	86.64
Rock Climbing	71	27	8	29.63	28	10	35.71
Rounders	272	190	107	56.32	263	193	73.38
Rowing	33	19	7	36.84	19	6	31.58
Rugby League	53	51	14	27.45	32	8	25
Rugby Union	234	229	208	90.83	151	82	54.3
Skiing	40	6	0	0	6	1	16.67
Soccer	279	267	227	85.02	251	159	63.35
Swimming	131	73	55	75.34	74	52	70.27
Table Tennis	216	203	92	45.32	194	81	41.75
Taekwondo	10	2	0	0	2	0	0
Tag / Touch Rugby	149	113	47	41.59	129	50	38.76
Tennis	222	199	118	59.3	209	114	54.55
Trampolining	182	141	70	49.65	166	88	53.01
Volleyball	200	168	78	46.43	180	82	45.56

When considering the number of schools that made an activity compulsory as a percentage of the number of schools that deliver that activity, the top activities for boys were: Rugby Union (90.83%), soccer (85.02%), athletics (79.70%), swimming (75.34%), cricket (75.10%), fitness (73.18%), gymnastics (72.54%), basketball (68.5%), badminton (63.27%), and dance (62.35%). For girls, the top activities were: judo (100%), netball (86.64%), athletics (76.67%), rounders (73.38%), gymnastics (73.15%), fitness (71.70%), swimming (70.27%), dance (67.09%), field hockey (65.15%), and soccer (63.35%).

### Perceptions of Risk

In 234 of the 288 schools', Subject Leaders for Physical Education voluntarily identified the activity that they delivered with the perceived highest risk of harm and injury, giving a total of 25 different activities (see Table 2). Here, 43% of Subject Leaders (n = 100) listed multiple activities as having the perceived highest risk of harm, providing a total of 334 responses. In rank order, these activities were: Rugby Union (*n* = 134, 67%), trampolining (*n* = 59, 38%), skiing (*n* = 5, 24%), soccer (*n* = 35, 15%), and field hockey (*n* = 25, 15%).

**Table 2:** The number of subject leaders that perceived an activity to be the riskiest activity of the activities their school delivered.

**Activity**	**Subject leaders that perceived as**		
	**the highest risk (of 234 schools)**		
	***N* that deliver**	***N***	**%**
Athletics	230	13	6
Basketball	222	14	6
Canoeing	24	1	4
Cycling	42	2	5
Dodgeball	167	3	2
Gaelic Football	28	1	4
Gymnastics	187	17	9
Handball	159	3	2
Hockey	171	25	15
Judo	26	1	4
Netball	216	11	5
Rock Climbing	67	2	3
Rounders	221	3	1
Rugby League	51	2	4
Rugby Union	201	134	67
Skiing	21	5	24
Soccer	228	35	15
Swimming	110	2	2
Trampolining	157	59	38
Volleyball	163	1	1

## Discussion

There is limited research about the composition of activities within the physical education curriculum (Smith et al., 2009; Whigham et al., 2019). This descriptive cross-sectional study was conducted to establish the extent to which individual activities were compulsory within state-funded secondary school physical education and to understand which activities Subject Leaders for Physical Education perceived to present the highest risk of harm. This study does not answer the question of which activities have the highest risk of injury and further research is warranted here.

In our study, Rugby Union was the tenth most delivered activity in state-funded secondary school physical education overall (*n* = 234, 81%), the eight most delivered activity for boys (*n* = 229, 83%), and the eighteenth most delivered activity for girls (*n* = 151, 54%). Thus, just over four-fifths of state-funded secondary school boys and one-half of secondary school girls were exposed to Rugby Union within their secondary school physical education.

### Perceived Risk of Harm

Subject Leaders for Physical Education identified the activities perceived to carry the highest risk of harm in this context. Given the lack of injury monitoring and epidemiological data available in physical education, such findings offer a valuable insight into injury risk in physical education settings. Here, Rugby Union was perceived to be the highest risk activity, both in terms of the number of Subject Leaders who selected this activity as carrying the highest risk of harm (*n* = 134) and as a proportion of schools that delivered the activity (*n* = 134, 67%). The perceived risk of injury was also substantially higher than the next perceived high-risk activity, namely trampolining (*n* = 59, 38%). For the sake of contrast, it is important to recognize that trampolining, as a product of its injury risk, requires teachers to hold sport-specific time-limited qualifications in order to facilitate the activity (Association for Physical Education, 2016). On the other hand, teachers can currently deliver Rugby Union without any sport-specific qualifications or training in English schools, despite Rugby Union having a relatively high risk of injury compared to other team sports (Roberts et al., 2013).

While this study did not examine how perceived risk translates to actual risk, these perceptions of activities that have a high risk of harm by Subject Leaders for Physical Education are in-line with epidemiological data from across sport, including hospital admissions data (Abernethy and MacAuley, 2003; Kirkwood et al., 2019) and concussion data (Pfister et al., 2016). Here, Kirkwood et al. found that football (soccer), Rugby Union, and Rugby League (respectively) resulted in the most sport-related hospital admissions for males under the age of 19 years, while Abernethy and MacAuley (2003) found contact rugby, including school games, physical education classes and competitive school matches, to be responsible for 44% of school-sport injuries that required Accident and Emergency department attendance.

### Compulsion

Within the state-funded secondary school physical education curriculum, a range of sporting activities were made compulsory by schools. Specifically, after soccer and athletics, Rugby Union was the third-highest activity that was made compulsory for boys (208 of 229 schools, 91%). Comparatively, Rugby Union was delivered less for girls (151 of 282 schools), with fewer schools also making this activity compulsory for girls (82 of 151 schools, 54%).

Given that Rugby Union has a relatively high risk of injury compared to other team sports (Roberts et al., 2013), strategies should be employed to mitigate this level of risk. Mitigation could be achieved through: (a) communication with children and parent(s) / guardian(s) concerning the short and long-term risks associated with participation in rugby; (b) acceptance of the risk by both the child and their parent(s) / guardian(s), of which non-volition on any party is the default position (White and Robinson, 2018); and (c) mandatory concussion training, Rugby Union specific time-limited qualifications and CPD for teachers, as is implemented in other leading rugby nations (Gianotti et al., 2009; Viljoen and Patricios, 2012). However, it is also essential to highlight that informed consent does not reduce the need for schools (and community sports clubs) to take every action possible to maintain safe practice.

Should there be a requirement for children and their parent(s) and guardian(s) to give their informed consent to engage in contact sports such as Rugby Union in physical education, further logistical challenges may arise in this context. For instance, some participants may give the appropriate permissions to participate in contact rugby while others might not, resulting in logistical challenges for how the teacher could appropriately supervise and manage two separate groups in the school physical education context. This may be most pronounced in the state-funded secondary school context, where there are often limited teaching resources in comparison to the independent school sector, alongside additional timetabling constraints.

As such, given the duty of care that schools must implement, the comparatively high risk of harm of Rugby Union, the lack of risk information presented to children and their carers, the lack of mandatory training for teachers, as well as the perceptions by Subject Leaders for Physical Education that Rugby Union is the riskiest sport, we argue that Rugby Union should not be a compulsory activity in school physical education. Instead, Rugby Union could exist as an opt-in (e.g., extra-curricular) activity for those who want to participate and who have accepted the risk of injury in doing so, or be replaced by non-contact codes of the game as recommended elsewhere (Cantu and Hyman, 2012). While more research is clearly needed on this topic until such data exists the precautionary principle should be applied, which would minimize the potential for harm to children in school physical education. Indeed, “The most effective… method for preventing concussion would be to eliminate exposure by removing the tackle from the game” (Cross et al., 2019).

### Study Limitations

This study offers some insight into those activities delivered within state-funded physical education in England. However, it does not show how many hours pupils are exposed to these activities within the physical education curriculum. Further research should explore the exposure time secondary-aged pupils have to each activity within the physical education curriculum in order to determine relative risk.

Subsequent research may also seek to capture what defines each of the identified activities beyond them being offered as part of the curriculum. Indeed, it is likely that activities within physical education classes will range from closed technical skills to contested match-play, with the associated injury risk differing according to the exposure of pupils to potentially high-risk events within those activities. Such research might provide a more comprehensive view on the nature of injury risk in curricular physical education.

Additional research could also seek to collect data from the other 37 counties in England which would result in a truly heterogeneous sample. Comparisons might then be made between schools based on school type, Ofsted rating, etc. where appropriate. At the same time, large prospective studies into injury surveillance in physical education are needed.

Currently, there is a lack of injury monitoring within school physical education. This means that the true incidence, prevalence and severity of injuries are unknown within this context. Health and Safety Executive guidance states that “If an accident that results in an injury arises because of the normal rough and tumble of a game, the accident and resulting injury would not be reportable.” As such, many injuries caused through participation in school physical education are not systematically recorded or available (Freitag et al., 2015). Mandatory reporting of injuries would allow for a more positivistic representation of injury risk in the physical education context than is available in this study.

## Conclusion

Rugby Union was delivered in physical education in 81% of the state-funded secondary schools we studied. Despite being perceived by Subject Leaders for Physical Education to be the activity with the highest risk of harm, Rugby Union was a compulsory activity for boys in 91% of those schools that deliver this activity as part of the curriculum, as well as being a compulsory activity for 54% of girls. Data from the amateur adult community context, youth community context, and after-school rugby settings also suggests that Rugby Union carries a relatively “high risk” of injury when compared to the other sports frequently delivered in school physical education.

Tackling is also argued to be unnecessary, leading us to contend that it should be reconsidered as a compulsory activity for children in physical education. Should young people need (or want) to be exposed to Rugby Union (or Rugby League) within the physical education curriculum, a non-contact version of the game could be employed. Those who continue to deliver tackle rugby should provide children and their parent(s) or guardian(s) with appropriate impartial information to give informed consent to participate.

## Data Availability Statement

The raw data supporting the conclusions of this article will be made available by the authors, without undue reservation.

## Ethics Statement

This study was reviewed and approved by the University of Winchester. The patients/participants provided their written informed consent to participate in this study.

## Author Contributions

AW, JB, NH, EA, RM, JP, PM, KP, ML, RB, AJP, LM, GT, JH, CH, GK, and AP have been involved in the conception of the research, involved in the acquisition, analysis and presentation of the data, contributed to the drafting, and editing of this manuscript. All authors contributed to the article and approved the submitted version.

## Conflict of Interest

The authors declare that the research was conducted in the absence of any commercial or financial relationships that could be construed as a potential conflict of interest.

## Publisher's Note

All claims expressed in this article are solely those of the authors and do not necessarily represent those of their affiliated organizations, or those of the publisher, the editors and the reviewers. Any product that may be evaluated in this article, or claim that may be made by its manufacturer, is not guaranteed or endorsed by the publisher.

## Footnotes

^1^Data obtained from Stokes K. (2020) WR Transgender Meeting - Stokes. https://playerwelfare.worldrugby.org/?subsection=84&fbclid=IwAR2v6U1YEgoH 1JIuGIe3ONmmWrdGW2lcV2fzXCMQfrKVjOGyu1dv8noIgZ8.

^2^Statistics obtained from the Department for Education (2017). Schools, pupils and their characteristics. Available online at: https://www.gov.uk/government/statistics/schools-pupils-and-their-characteristics-january-2017 (accessed March 21, 2020).
